# From Physicochemical Properties to Rehabilitation Outcomes: Understanding Corticosteroid Injection Adverse Effects

**DOI:** 10.3390/ijms27167417

**Published:** 2026-08-19

**Authors:** Carmelo Pirri, Nicola Manocchio, Andrea Sorbino, Valeria Napoleoni, Anna D’Amato, Nina Pirri, Calogero Foti

**Affiliations:** 1 Department of Neurosciences, Institute of Human Anatomy, University of Padova, 35121 Padova, Italy; 2 Physical and Rehabilitation Medicine, Department of Clinical Sciences and Translational Medicine, Tor Vergata University, 00133 Rome, Italy; a.sorbino@quaderni.biz (A.S.); valeria.nap2@gmail.com (V.N.); damatoanna.92@gmail.com (A.D.); foti@med.uniroma2.it (C.F.); 3 Department of Radiology, Ospedale Santa Maria della Misericordia, 45100 Rovigo, Italy; nina_92_@hotmail.it

**Keywords:** corticosteroids, injections, intra-articular, musculoskeletal diseases, rehabilitation, ultrasound imaging

## Abstract

Corticosteroid injections (CSIs) are widely used in Physical and Rehabilitation Medicine, but their safety profile is strongly influenced by the formulation-specific chemical structure and physicochemical properties of each molecule, as well as by injection technique and dosing strategy. Particulate corticosteroids such as triamcinolone acetonide and methylprednisolone acetate exhibit low aqueous solubility, microcrystal formation, and prolonged intra-articular residence, which translate into sustained anti-inflammatory effects but also higher risks of chondrotoxicity, calcifications, tendinopathy, cutaneous and muscular atrophy, osteonecrosis, nerve injury, infection, and post-injection flare, especially with repeated or high-dose use. In contrast, more soluble preparations like betamethasone and dexamethasone sodium phosphate provide rapid onset and shorter duration of action, with reduced local depot-related complications but a greater propensity for transient systemic effects such as glycemic spikes. Across adverse events, less soluble, longer-acting formulations and inaccurate extra-articular delivery consistently emerge as key drivers of local tissue damage. Ultrasound guidance significantly improves injection accuracy, optimizes drug deposition of both particulate and non-particulate agents, and may enhance clinical outcomes while limiting complications, thereby representing a relevant component of CSI practice. In conclusion, this narrative review proposes a formulation-oriented framework for corticosteroid selection, integrating pharmaceutical formulation, physicochemical properties, imaging guidance, and individualized rehabilitation strategies to optimize molecule selection, minimize adverse effects, and advance a precision rehabilitation paradigm.

## 1. Introduction

Corticosteroids are adrenal steroid hormones that play a central role in the regulation of inflammation, immune responses, metabolism, and fluid–electrolyte balance [1]. They exert their effects mainly through glucocorticoid and mineralocorticoid receptors, modulating the transcription of numerous genes involved in inflammatory and metabolic pathways [2,3]. In clinical practice, the term ‘corticosteroids’ usually refers to glucocorticoids, which are widely used for their potent anti-inflammatory and immunosuppressive actions [4].

In Physical and Rehabilitation Medicine (PRM), corticosteroid injections (CSI) are frequently employed to manage pain and inflammation in musculoskeletal conditions such as tendinopathies, bursitis, and osteoarthritis (OA), facilitating participation in individual rehabilitation projects [5]. However, CSI are typically used as a short-term intervention, providing pain relief for weeks to months; they do not address the underlying cause of the condition [6]. Moreover, the safety profile of CSI appears to be strongly influenced by the specific molecule, its physicochemical and formulation-specific chemical structure, the dose, and the injection technique [7,8].

Wang and Hutchinson, in a study of 18 diabetic patients, reported an average increase of 73% in blood glucose levels on the first morning after CSI [9]. Conversely, a clinical study conducted by Catalano et al. also found a statistically significant increase, but the authors concluded that it was not clinically meaningful, with mean increases of 14.2 and 9.7 mg/dL on days 1 and 5 after injection [10]. However, the literature emphasizes the need for blood glucose monitoring in the days following CSI, especially in patients with poor metabolic control or on insulin therapy [11,12,13]. Lastly, the American Diabetes Association also recommends caution and glycemic monitoring during glucocorticoid therapy, including local administrations, due to the risk of hyperglycemia and related complications [14].

The most frequently reported side effects include localized reactions at the injection site. Injection site pain, discomfort, erythema, induration, and edema are commonly noted [15,16]. In some cases, these reactions can contribute to a temporary reduced range of motion in the affected area [17]. While acute side effects are prevalent, repeated CSI can lead to delayed and potentially more severe complications. There is evidence supporting a clear association between frequent CSI and complications such as skin atrophy, striae, and hypopigmentation, reflecting the impact of corticosteroids on dermal structures [18]. Concerns regarding tendon weakening and subsequent atrophy have been documented, as corticosteroids can impair collagen synthesis and affect tendon integrity. Repeated use can lead to tendon degeneration, potentially delaying recovery and rehabilitation processes for patients undergoing treatment [19].

The dosage and technique of injection significantly influence these risks. Improper techniques, particularly intratendinous needle placement, can lead to deleterious mechanical effects, increasing the risks associated with CSI [20]. The dosage of corticosteroids administered significantly impacts the overall safety profile of the procedure. Higher dosages and closer injection intervals have been correlated with an increased risk of adverse effects such as soft tissue atrophy [21]. The selection of corticosteroids, guided by specific clinical contexts and patient conditions, is essential in minimizing adverse outcomes.

The proper choice of corticosteroid molecules and dosage necessitates a robust foundation of evidence from the literature, which is presently insufficient. Consequently, this narrative review aims to synthesize current evidence on adverse events associated with CSI, highlighting how formulation-specific chemical structure, physicochemical properties, and drug choice may impact chondrotoxicity, tendinopathy, osteonecrosis, calcifications, and systemic side effects in a rehabilitation context.

## 2. Methods

This article was designed as a narrative review rather than a systematic or scoping review. The primary objective was to provide a comprehensive and clinically oriented synthesis of current evidence on the adverse effects of corticosteroid injections in musculoskeletal and rehabilitation practice, with particular attention to the influence of molecular properties, dose, and injection technique. To this end, we performed a non-systematic search of the literature in major scientific databases (including PubMed, Scopus and Web of Science), focusing on publications relevant to corticosteroid pharmacology and to key adverse events such as chondrotoxicity, tendinopathy, osteonecrosis, calcifications, and systemic metabolic effects. The search was not restricted to a predefined time window, in order to capture both seminal historical studies and more recent investigations. Keywords, alone or in combination, included terms such as ‘corticosteroid injections’, ‘glucocorticoids’, ‘triamcinolone’, ‘methylprednisolone’, ‘betamethasone’, ‘dexamethasone’, ‘adverse effects’, ‘chondrotoxicity’, ‘tendinopathy’, ‘osteonecrosis’, ‘calcification’, and ‘rehabilitation’. No formal risk-of-bias assessment or quantitative synthesis was performed, consistent with the narrative nature of the review.

## 3. Pharmacological Aspects of Most Commonly Used Corticosteroids in Rehabilitation

From a pharmacological perspective, injectable corticosteroids used in musculoskeletal rehabilitative practice differ in their chemical structure, physicochemical properties, pharmaceutical formulation, solubility, microcrystal behavior, receptor-binding affinity, enzymatic degradability, and systemic exposure, and these differences determine their clinical application. Particulate corticosteroids, in particular triamcinolone acetonide (TA) and methylprednisolone acetate (MPA), are characterized by low aqueous solubility and structural features that favor crystalline depot formation. Methylprednisolone acetate contains an acetate ester at the C21 hydroxyl group, whereas triamcinolone acetonide contains a cyclic acetonide bridging the C16 and C17 hydroxyl groups. These microcrystals dissolve slowly in synovial fluid, producing prolonged intra-tissue retention and a sustained release kinetic, with pharmacokinetic studies demonstrating measurable synovial persistence for several days and extended cortisol suppression for 2–4 weeks [22]. Among particulate corticosteroids, triamcinolone acetonide exhibits greater lipophilicity than methylprednisolone acetate, which may contribute to its slower dissolution and prolonged tissue persistence. These physicochemical characteristics may contribute to the longer duration of clinical benefit reported for triamcinolone acetonide compared with methylprednisolone acetate in selected conditions such as osteoarthritis and adhesive capsulitis [23,24].

Pharmacodynamically, particulate corticosteroids produce a slow, steady intracellular accumulation of the active moiety following enzymatic de-esterification by local esterases. This gradual liberation creates a low-fluctuation glucocorticoid receptor occupancy pattern, which correlates with a smoother anti-inflammatory effect and more stable symptom control. Their crystalline nature, however, also generates a higher risk of post-injection flare, a transient inflammatory reaction triggered by crystal–synovial membrane interactions and mild mechanical irritation. Additionally, particulate agents show stronger depot behavior, with drug microreservoirs physically embedded within the synovial folds and periarticular fat pads, contributing to long-acting profiles but also to delayed systemic absorption.

In contrast, highly water-soluble corticosteroid formulations, particularly dexamethasone sodium phosphate and betamethasone sodium phosphate, exhibit high aqueous solubility due to phosphate esterification at C21, which introduces a strongly polar, ionizable phosphate group. From a physicochemical perspective, this modification prevents crystal formation and enables rapid dissolution following injection. Their pharmacokinetic profile is characterized by rapid absorption, early peak plasma concentrations, and shorter intra-articular residence, producing a therapeutic effect that is rapid in onset but shorter in duration. Following injection, these phosphate ester formulations undergo rapid hydrolysis by tissue phosphatases, releasing the dexamethasone or betamethasone, which are subsequently redistributed systemically.

This pharmacological profile makes them particularly suitable for acute inflammatory flares, soft-tissue injections, or anatomical regions where prolonged corticosteroid persistence may increase the risk of local tissue injury, such as peritendinous regions, small joints, and perineural applications.

**Figure 1:**
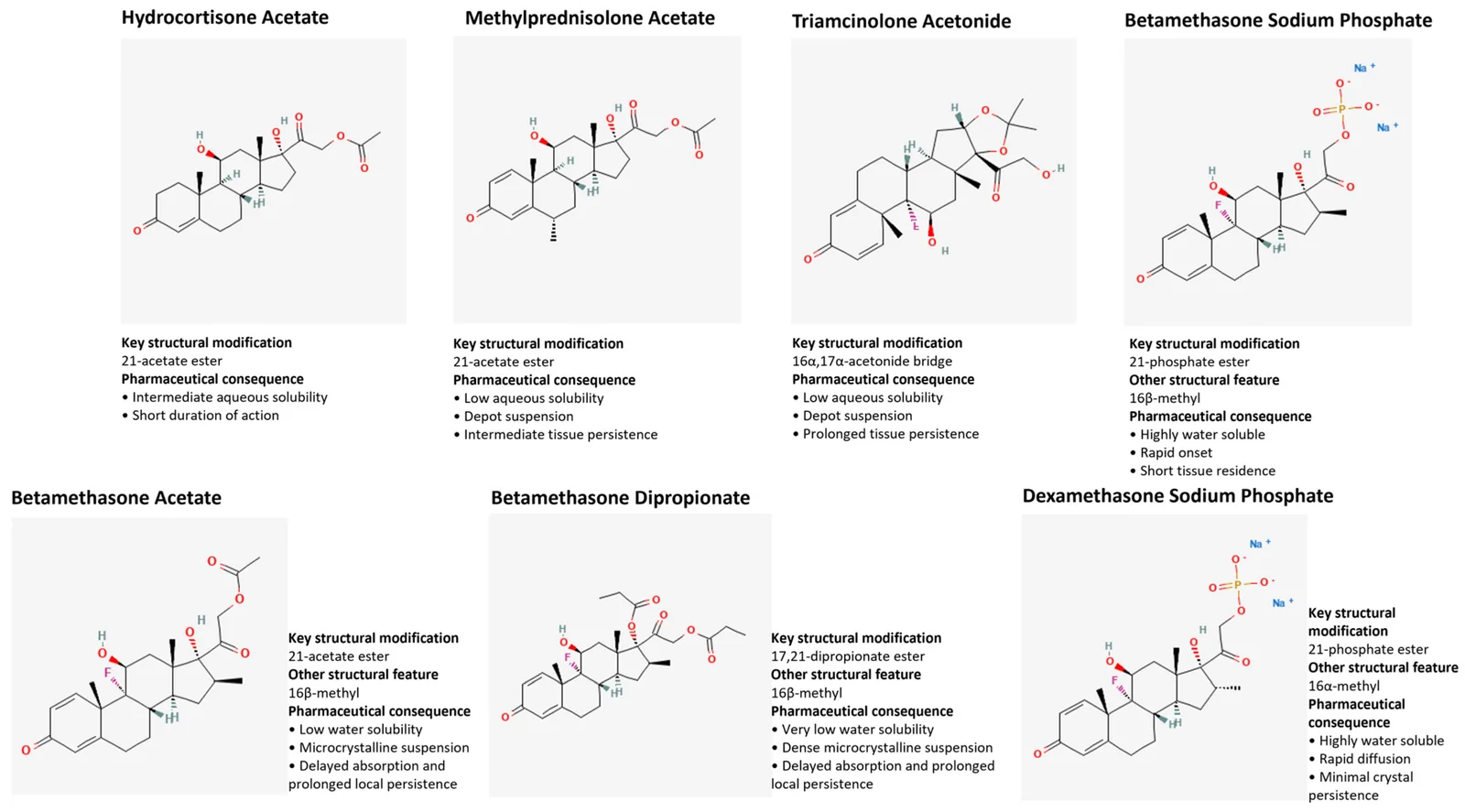
Structural comparison of the injectable corticosteroid formulations most commonly used in rehabilitation. The figure highlights the principal structural modifications defining each pharmaceutical formulation together with its expected physicochemical and pharmacological consequences. Chemical structures were obtained directly from the PubChem Compound database and assembled by the authors.

Among the highly soluble phosphate formulations, dexamethasone sodium phosphate and betamethasone sodium phosphate retain the high intrinsic glucocorticoid receptor affinity of their parent corticosteroid molecules. Their high anti-inflammatory potency is primarily attributable to the molecular structure of dexamethasone and betamethasone, which share 9α-fluorination and C16 methyl substitution but differ in the stereochemical orientation of the C16 methyl group, being 16β in betamethasone and 16α in dexamethasone. Their sodium phosphate formulations additionally contain a phosphate ester at C21, which primarily enhances aqueous solubility and determines their pharmaceutical behavior rather than intrinsic receptor affinity. These structural features enhance glucocorticoid receptor affinity while minimizing mineralocorticoid activity. These molecular features provide potent anti-inflammatory effects at relatively low doses by promoting rapid glucocorticoid receptor activation, nuclear translocation, and effective transcriptional suppression of NF-κB and AP-1 signaling pathways, although without the prolonged intra-articular residence characteristic of particulate depot formulations. Hydrocortisone acetate represents a distinct formulation with intermediate physicochemical characteristics. Although less water-soluble than phosphate ester formulations, it demonstrates substantially shorter tissue persistence and lower glucocorticoid receptor affinity than triamcinolone acetonide or methylprednisolone acetate. Consequently, its clinical duration of action is comparatively limited, making it appropriate when a shorter corticosteroid effect or lower cumulative glucocorticoid exposure is desirable (Figure 1).

These pharmacological divergences translate directly into distinct kinetic and metabolic behaviors. Particulate corticosteroids undergo slow, multi-phase release governed by crystal erosion, esterase-mediated hydrolysis, and gradual diffusion into systemic circulation. This pharmacokinetic architecture results in flat concentration–time curves, reduced peak exposure, and prolonged—but more predictable—HPA-axis suppression. Soluble corticosteroids, in contrast, generate steep concentration–time curves, with high initial plasma levels, stronger but shorter HPA-axis suppression, and quicker return to physiological homeostasis. Clinically, this means that soluble steroids carry a higher risk of transient systemic side effects (e.g., glycemic spikes), whereas particulate steroids carry a greater risk of cumulative exposure with repeated injections.

Clinically, these differences align strongly with indication patterns: particulate agents such as TA and MPA are ideal for large joints (knee, shoulder, hip), chronic synovitis, adhesive capsulitis, degenerative arthropathies, and conditions requiring long-lasting anti-inflammatory control. Soluble agents are preferred for hand and wrist joints, tenosynovitis, bursal pathology, acute inflammatory flares, and in situations where rapid onset with minimal local persistence is therapeutically advantageous. Sonographically, particulate steroids appear as bright, echogenic microcrystal clouds, while soluble steroids disperse as homogeneous anechoic solutions, reflecting their distinct physicochemical profiles [25].

In summary, the pharmacological behavior of intra-articular corticosteroids is dictated by the interplay of formulation-specific chemical structure, physicochemical properties, solubility, molecular modifications, enzyme susceptibility and receptor-binding dynamics (Table 1). These factors collectively shape dissolution rate, tissue residency, systemic bioavailability, potency, safety and ultimately clinical utility. A precise understanding of these mechanistic distinctions is essential for tailoring steroid selection to specific musculoskeletal conditions with maximal therapeutic precision. Rather than considering injectable corticosteroids as therapeutically interchangeable agents, the evidence reviewed in this manuscript supports a formulation-oriented approach in which pharmaceutical formulation, crystal behavior, solubility, tissue persistence and release kinetics become integral determinants of clinical decision-making. This links molecular characteristics to anatomical target, expected tissue response, and rehabilitation objectives, providing a mechanistic rationale for personalized corticosteroid selections in musculoskeletal rehabilitation.

## 4. Chondrotoxicity

Corticosteroids exhibit a time- and dose-dependent influence on articular cartilage. Low doses and short durations can have beneficial effects, whereas high doses (>3 mg/dose or 18–24 mg/dose cumulative total) and prolonged exposure demonstrate detrimental degradative effects. Specifically, methylprednisolone, dexamethasone sodium phosphate, and betamethasone sodium phosphate at these higher regimens interfere with the mechanisms involved in cartilage protein production, notably impacting proteoglycans, type II collagen, and aggrecan synthesis [26].

The chondrotoxic effects of intra-articular CSI can be linked to an accelerated progression of OA [27]. This acceleration is characterized by a rate of joint space loss that exceeds the typical progression observed over a short timeframe [28].

Zeng et al. reported a significantly elevated probability of radiographic OA progression in patients receiving intra-articular corticosteroid injections in the knee joint. Their findings indicated a 3.2-fold increased likelihood of progression compared to non-injected patients. Notably, this probability was even higher (4.67 times) in patients who received continuous CSI. This suggests a correlation between intra-articular corticosteroid administration, particularly repeated injections, and an increased risk of OA advancement as observed through radiographic measures [29]. Radiographic progression of OA following injection has also been demonstrated in the hip: one study reported radiographic progression of hip joint space narrowing in 44% of patients who received injections in the hip (40 mg triamcinolone/4 mL 0.5%) and were followed post-procedure for an average of 6 months [30]. Relevant evidence on the chondrotoxic potential of corticosteroids has been reported in a scoping review by Pirri et al. [31]: 58% of the reviewed studies showed possible chondrotoxic effects of corticosteroids, raising concerns about their clinical use. Betamethasone demonstrated signs of toxicity both in vivo (in rabbits and dogs) and in vitro, with reduced proteoglycan levels and macroscopic cartilage damage. Only one study did not report harmful effects. Methylprednisolone was also widely studied, with ten studies generally reporting negative results: reduced cell viability, cartilage damage, and enhanced toxicity when combined with lidocaine. However, some studies found no adverse effects, and one suggested potential structure-modifying activity on joints. Regarding triamcinolone, findings were mixed: some studies showed negative effects (oxidative stress, cell death, altered cartilage metabolism), while others found no damage or even protective effects on chondrocytes. Dexamethasone was evaluated in five studies, four of which reported harmful outcomes (apoptosis, inhibition of extracellular matrix synthesis, suppression of remodeling factors). Only one study showed a reduction in inflammatory mediator expression. Lastly, three studies focused on hydrocortisone: one reported cartilage damage, while two in vitro studies observed beneficial effects, such as enhanced extracellular matrix synthesis and inhibition of degenerative enzymes. Among the most widely used depot glucocorticoids in clinical practice is betamethasone acetate, a sustained-release preparation characterized by a poorly soluble acetate component that prolongs its intra-articular permanence. In vitro studies on human chondrocytes have demonstrated that, unlike dexamethasone sodium phosphate and methylprednisolone acetate, this combination induces significant and consistent chondrotoxicity both after exposure for the mean duration of action (approximately 9 days) and in a 14-day time-controlled trial, with cell mortality exceeding 20%, well above that observed with the other corticosteroids tested. The authors hypothesize that this toxicity is linked both to benzalkonium chloride, a preservative present in the formulation, and to the crystalline structure of betamethasone acetate, physically similar to the monosodium urate crystals found in gout, capable of triggering chondrocyte damage mediated by inflammatory-like mechanisms [32]. The association of betamethasone acetate with local anesthetics, a common practice for post-injection pain control, further amplifies this toxic effect. In a bioreactor model reproducing synovial fluid metabolism, the combination of betamethasone with 1% lidocaine or 0.25% bupivacaine resulted in dramatic chondrocyte mortality of 76% and 66% at 14 days, respectively, significantly higher than both controls and local anesthetics alone (Braun et al. 2012 [33]). This synergistic effect was markedly more pronounced than that observed with the other combinations tested (dexamethasone, methylprednisolone acetate, triamcinolone acetonide with the same anesthetics), leading the authors to recommend particular caution in the combined use of insoluble betamethasone and intra-articular local anesthetics [33].

CSI may also cause chondrolysis, a condition characterized by the rapid and progressive destruction of articular cartilage, leading to significant joint pain and stiffness [34]. Overt chondrolysis following a single CSI is rare; however, the risk increases when corticosteroids are combined with local anesthetics, particularly lidocaine and bupivacaine, which are known for their chondrotoxic properties [23,35]. According to both experimental and clinical evidence, triamcinolone is the most extensively studied corticosteroid. Clinical trials demonstrated that repeated intra-articular injections of triamcinolone (40 mg every three months for two years) may be associated with a significantly greater reduction in cartilage volume compared to placebo, suggesting a potential risk of chondrolysis or accelerated cartilage degeneration [24,36]. At the time being, the overall safety of intra-articular corticosteroids remains suboptimal, particularly with repeated administration or when combined with local anesthetics (Table 2).

## 5. Calcification

Calcification is one of the most common side effects of CSI [37]. These calcifications are mostly pericapsular or intracapsular, and rarely intra-articular, composed of hydroxyapatite. The location of the calcification is related to the site of the injection and varies in terms of size, shape, and structure [38]. The causal mechanism is related to local tissue damage caused by the needle and the low water solubility of the extra-articular corticosteroid, which leads to chronic granulomatous inflammation and subsequent dystrophic calcification [39]. Intra-articular CSIs of triamcinolone hexacetonide are a useful therapy for chronic OA in children; youth and joint size are possible predisposing factors for subcutaneous tissue atrophy and intra-articular calcification [40,41]. Furthermore, extensive calcification of a spinal synovial cyst has been reported after an intra-articular injection of a long-acting corticosteroid [42]. Additionally, the development of a synovial cyst was documented in a 57-year-old man following an intra-facet joint injection of prednisone, with subsequent calcification observed six months post-injection [43]. Soft tissue calcification directly concerns insoluble betamethasone, as the combination of betamethasone sodium phosphate and betamethasone dipropionate is expressly indicated as the agent of choice for peri-articular injection and for injection at the origin/insertion of muscles and bursae, i.e., the same anatomical sites for which the risk of calcification is reported. Chinese expert panel guidelines therefore recommend paying particular attention to the perceived resistance during peri-musculotendinous and peri-ligament injection with betamethasone dipropionate, avoiding direct intratendinous injections, as a key preventive measure against this complication [44] (Table 3).

## 6. Tendinopathy

The literature suggests that tendon rupture following CSI may be attributed to several mechanisms, including impaired collagen synthesis, cytotoxic effects on tenocytes, reduced tendon vascularity, and biomechanical weakening, increasing the tendon’s susceptibility to rupture [45]. Specifically, triamcinolone has been hypothesized to directly harm human tenocytes by suppressing their cellular activity and collagen production [46], potentially leading to altered tendon structure and increased risk of spontaneous rupture [47]. Triamcinolone acetonide has been shown to reduce proteoglycan synthesis, while dexamethasone inhibits tendon cell migration [45,48]. In vitro studies on rat and human tendon cells have demonstrated that dexamethasone can inhibit cell proliferation and reduce collagen synthesis [49]. Wen-Chung Tsai et al. conducted a study on Achilles tendon fibroblasts and demonstrated that an initial inhibitory effect on the proliferation was observed at a concentration of 10–4 M of dexamethasone [50].

An in vivo study on rats showed that both triamcinolone acetonide and prednisolone significantly reduced the biomechanical strength of Achilles tendons, with histological analysis revealing collagen attenuation, increased expression of MMP-3, and apoptotic cells in the corticosteroid-treated groups [51]. Similar findings in rotator cuff tendons indicate that triamcinolone acetonide decreases proliferation rates, collagen synthesis, and tendon cell migration by modulating MMP2, MMP9, and TIMP1 expression [46,52].

Corticosteroid preparations for injection often contain microcrystalline suspensions designed for sustained release at the injection site. While effective for managing inflammation, these microcrystals can sometimes precipitate at the injection site, potentially causing local irritation or a post-injection flare [53]. Microcrystals may trigger localized necrosis and degenerative changes if injected directly into tendon tissue, weakening tensile strength and predisposing to tendon rupture. Moreover, corticosteroid microcrystals can physically shield bacteria from immune defenses in the area, increasing the risk of local infection after injection [54]. Triamcinolone formulations (especially acetonide and hexacetonide) have large, persistent microcrystals that last longer in tissues and can aggregate substantially. Methylprednisolone acetate also tends to form sizable aggregates and is associated with a higher risk of tissue reactions and tendon damage, similar to triamcinolone [55]. Betamethasone is less particulate, its crystal size is usually smaller, and it is less likely to persist and accumulate than triamcinolone or methylprednisolone [56]. In comparative studies of tendon lesions and intra-articular injections, triamcinolone and methylprednisolone were both linked to a greater risk of tendon rupture than betamethasone [57].

Compound betamethasone (betamethasone sodium phosphate combined with the poorly soluble dipropionate/acetate ester) is widely used for tendinopathic and peritendinous pain, where its dual rapid-onset/long-acting profile is considered advantageous: in patients with persistent pain after supraspinatus tendon repair, local blocking therapy with compound betamethasone significantly improved pain, sleep quality, and shoulder function versus controls [58], and it has similarly served as the corticosteroid comparator in trials of rotator cuff impingement, producing significant short-term benefit comparable to platelet-rich plasma at three months, though a meta-analysis of PRP versus corticosteroids (including betamethasone) found no significant difference in short-term pain relief and a non-significant trend favoring PRP for function beyond six months [59]. However, its particulate acetate component carries the same tendon-safety concerns described for other insoluble corticosteroids, being implicated in collagen-synthesis suppression and tendon rupture, particularly with injections performed within six months of tendon repair surgery or with repeated dosing [28]; in De Quervain tendinopathy, a single betamethasone sodium phosphate/acetate injection achieved a 70% success rate with clinically meaningful DASH and VAS improvement, though anatomical variants such as an intracompartmental septum markedly increased recurrence risk, with adverse events limited to minor, self-resolving skin changes [60] (Table 4).

## 7. Osteonecrosis

Osteonecrosis, also referred to as avascular necrosis, ischemic necrosis, osteochondritis dissecans, and aseptic necrosis, is a serious condition characterized by the death of bone tissue due to a lack of blood supply. Non-traumatic osteonecrosis has been linked to various natural and iatrogenic factors, including congenital conditions such as Legg-Calvé-Perthes disease and chronic use of medications like corticosteroids. The femoral head is the most commonly affected site, though other bones can also be involved [61]. The pathogenesis of osteonecrosis involves multiple mechanisms, but the final common pathway is the failure to deliver nutrients to the affected bone, leading to bone cell death. Early events may include vascular injury, mechanical stress, increased intraosseous pressure, adipocyte dysfunction, defects in apoptosis, and coagulation abnormalities. Ultimately, these processes converge to impair blood flow to the watershed areas of the femoral head, resulting in osteonecrosis [62].

Corticosteroid use is a well-recognized risk factor for non-traumatic osteonecrosis, particularly when administered in high doses or over extended periods. The exact mechanisms by which corticosteroids induce osteonecrosis are not fully understood, but several factors are implicated [63]. Corticosteroids can induce apoptosis of bone cells via pathways such as the Fas pathway, leading to increased cell death in bone tissue [64]. Steroids alter lipid metabolism, causing swelling and necrosis of adipocytes, increased lipid deposition in osteocytes, hyperlipidemia, fat embolism, and fatty infiltration of the bone marrow. These changes can obstruct blood vessels and impair bone perfusion [65,66]. Corticosteroids can damage vascular endothelial cells and promote a hypercoagulable state, increasing the risk of thrombus formation and further reducing blood supply to bone [67,68]. Steroid-induced oxidative injury may also contribute to the development of osteonecrosis [69,70,71]. Glucocorticoids inhibit osteoblast viability and function, impairing bone formation and repair [72,73]. A randomized trial used betamethasone dipropionate (10 mg) in combination with betamethasone sodium phosphate (4 mg) for the treatment of post-traumatic subtalar OA but did not report any adverse bone events during the 24-week follow-up, likely due to the short duration of the study and the small sample size [74] (Table 5).

## 8. Cutaneous Atrophy and Hypopigmentation

Cutaneous atrophy is a frequently underrecognized local adverse effect of CSI administered for musculoskeletal conditions. Patients who develop cutaneous atrophy following CSI may be misdiagnosed with disorders such as linear morphea, atrophoderma, or vascular diseases, which can lead to unnecessary diagnostic procedures and delays in appropriate treatment [21].

Clinically, corticosteroid-induced cutaneous atrophy typically presents as atrophic or scleroderma-like macules, often accompanied by hypopigmentation or depigmentation. These lesions characteristically display a linear or branching distribution pattern. Occasionally, erythematous or vascular features such as purpura and telangiectasias may be observed [75,76]. Most cases are asymptomatic, though some reports have noted associated paresthesia [77,78].

Histopathologically, these lesions are marked by epidermal atrophy without significant inflammation in the dermis. There is a notable decrease in melanin content, though the number of melanocytes is typically preserved [79,80]. The pathogenesis of atrophy involves corticosteroid-induced inhibition of keratinocyte and fibroblast proliferation, as well as alterations in lipid synthesis and protein metabolism in the skin [81]. Additionally, corticosteroids can cause local vasoconstriction, leading to thrombosis, hypoxia, and subsequent atrophy [79,82].

Microscopic examination of affected tissue reveals a reduction in both the number and size of adipocytes, often accompanied by infiltration of lipophage-like macrophages. Other inflammatory cells and tissue necrosis are generally absent. Skin biopsies demonstrate thinning of the epidermis and dermis, flattening of rete ridges, reduced melanin-containing cells, and homogenization of collagen fibers. Glucocorticoids have also been shown to decrease the synthesis of type I and type III collagen, contributing to dermal thinning [78,83].

The natural history of corticosteroid-induced soft tissue atrophy and hypopigmentation is relatively consistent. These changes typically manifest between 2 and 4 months after injection but may be delayed up to 10 months in some cases [84,85]. Atrophy is primarily due to loss of subcutaneous adipose tissue and decreased collagen production, resulting in thinning of the skin, while hypopigmentation is mainly attributed to melanocyte dysfunction [83]. Less soluble, longer-acting corticosteroid preparations are more likely to cause persistent soft tissue changes [86,87].

Skin hypopigmentation has been reported to occur in 1.3–4% of patients following local CSI [88]. While the precise mechanism of hypopigmentation remains unclear, it is understood that steroids or their biologically inactive components can contribute to this effect [89]. Dermal complications after corticosteroid injection may also arise from mechanical factors, such as edema, alterations in ground substances, or vasoconstriction. Hypopigmentation typically manifests 1–4 months post-injection [90]. The choice of steroid for injection is often guided by its solubility: steroids with low solubility, like triamcinolone acetonide, are generally preferred for injections into deep structures such as the knee, elbow, and shoulder joints, whereas steroids with high solubility, such as betamethasone sodium phosphate and dexamethasone sodium phosphate, are typically used for injections into soft tissues like the bursa, tendon sheath, metacarpophalangeal joint, proximal phalangeal joint, and carpal tunnel. Subcutaneous fat atrophy is another recognized complication, with a reported duration of 6–12 months post-corticosteroid injection, but it is generally reversible, resolving within one year [84].

In a prospective cohort study conducted specifically with betamethasone dipropionate/sodium phosphate in the dorsal wrist and elbow hypopigmentation appeared in 37.8% of cases (17/45), almost exclusively after wrist injections and in female patients, with onset between 4 and 10 weeks and complete resolution in an average time of 7.14 months, while subcutaneous fat atrophy, rarer (6.7% of cases), showed incomplete recovery in two out of three cases even after 18 months of follow-up [91]. The authors attribute the greater vulnerability of the wrist district, compared to the elbow, to the lower thickness of the subcutaneous tissue, and hypothesize that the particulate nature of betamethasone dipropionate contributes to amplifying the risk through a “depot” effect which prolongs the local exposure of the tissue to the drug. Consistently, a narrative review on intra-articular therapies for hand OA reports that the incidence of hypopigmentation and skin atrophy doubles with peri-articular injection (10%) compared to strictly intra-articular injection (4.7%), underlining the importance of the precision of the injection technique also for betamethasone acetate/sodium phosphate-based formulations commonly used in this anatomical site [92]. It should also be noted that a randomized comparative study on knee OA, which used betamethasone dipropionate/sodium phosphate compared with methylprednisolone acetate, did not record any cases of skin hypopigmentation or subcutaneous atrophy during the 12-week follow-up, suggesting that the incidence of this complication may vary significantly depending on the anatomical site of injection and the duration of observation [93] (Table 6).

## 9. Muscular Atrophy

Muscle weakness and atrophy are well-recognized complications of corticosteroid therapy and represent the most common form of drug-induced myopathy encountered in clinical settings [94]. The risk of developing corticosteroid-induced myopathy depends on the specific corticosteroid used, as well as the dose and duration of treatment. Prolonged therapy, especially with triamcinolone, betamethasone, and dexamethasone, is most strongly associated with symptomatic myopathy, though chronic administration of prednisone at doses greater than 10 mg per day, or equivalent doses of other corticosteroids, can also lead to this complication [95,96]. Electromyographic studies have demonstrated a high prevalence of subclinical myopathy in patients undergoing long-term oral corticosteroid therapy, even in the absence of overt symptoms [97]. Laboratory findings typically reveal normal serum creatine kinase and other muscle enzyme levels, while electromyography shows classic myopathic changes, including reduced duration and amplitude of motor unit action potentials without evidence of spontaneous electrical activity in the muscle. However, increased urinary creatine excretion is often observed [98,99]. Muscle biopsy in corticosteroid-induced myopathy typically reveals selective atrophy of type 2 muscle fibers without evidence of muscle fiber destruction, and may show increased neutral lipid content within muscle fibers [97]. On rare occasions, muscle atrophy has also been reported following CSI. Park et al. reported the case of a young woman who developed severe muscle atrophy in the wrist after a single injection of triamcinolone acetonide (20 mg) into the transverse carpal ligament [84]. Pre-clinical data suggest frequent CSI could increase the expression of some detrimental genes, such as muscle atrophy-related genes, supporting this notion [100]. In their in vitro study, Gartling et al. report data about thyroarytenoid muscle atrophy after Dexamethasone injections at the vocal fold. The authors’ findings are in agreement with the idea that CSI may alter local gene expression in a harmful way [101].

Compared to skin and subcutaneous atrophy, muscle tissue atrophy associated with betamethasone dipropionate/sodium phosphate injections is much less documented in the literature, likely due to the lower direct exposure of muscle tissue to the drug during common periarticular or intra-articular injections. In a randomized controlled trial using a composite solution of betamethasone sodium phosphate (1.25 mg) and betamethasone dipropionate (0.5 mg) in combination with lidocaine for the ultrasound-guided treatment of chronic rotator cuff tears, muscle atrophy was included among the adverse events actively monitored for the entire duration of the study (3 months), but no cases were reported or detected through patient logs and standardized questionnaires. This data, although negative, is nevertheless relevant for the safety profile of the formulation, since it suggests that the risk of clinically evident muscle atrophy is low in the short-medium term with this type of dosing regimen (three injections at weekly intervals), although the same authors underline that the short duration of the follow-up does not allow to exclude late effects linked to the persistence of the genomic action of corticosteroids, described as significantly longer than the pharmacological half-life of 36–54 h of the compound betamethasone [102] (Table 7).

## 10. Nerve Lesions

Nerve injury represents a notable risk associated with steroid injections, particularly in the context of CSI for conditions like carpal tunnel syndrome (CTS) [103]. Evidence indicates that repeated corticosteroid injections may not provide substantial long-term benefits and could potentially worsen symptoms through mechanical stress on peripheral nerves [104]. For instance, the case reported by Zhou and Lu documented severe median nerve injury following a local corticosteroid injection for CTS [105]. Their findings suggest a possible link between repeated CSI and increased nerve damage, potentially resulting from scar tissue proliferation at the nerve epineurium. Moreover, the safety profile of CSI is not without risks, as studies have shown that these injections can result in complications such as tendon damage and inflammation in the vicinity of nerves within the carpal tunnel. These complications can contribute to the persistence of symptoms following the injection [15]. Notably, one proposed mechanism for the efficacy of CSI in CTS is the decompression of the median nerve achieved through the thinning of the flexor tenosynovium. Consequently, this theory suggests that CSI may not offer any superior therapeutic benefits compared to other injectable substances that could similarly reduce tenosynovial thickness or volume, with concerns about their application [106]. Nerve injuries after CSI have also been reported in other body districts. Snow et al. reported a case of a 41-year-old man who developed pain and numbness in the lateral aspect of his foot after a steroid injection for plantar fasciitis. The examination and electromyographic studies confirmed damage to the lateral plantar nerve [107] (Table 8).

## 11. Risk of Infection

Chronic and short-term treatment with oral corticosteroids has been associated with an increased risk of infection. However, the potential risk of infection related to intra-articular and soft-tissue corticosteroid injections has not been well reported. Zacay and Heymann conducted a study aimed at assessing the risk of infection following intra-articular or soft-tissue corticosteroid injections [108]. The research was designed as a self-controlled risk interval study including 15,732 adults who received intra-articular or soft-tissue corticosteroid injections between 2015 and 2018. The researchers identified the occurrence of several common infections in patients’ electronic medical records and analyzed the incidence rates of all infections collectively, as well as each infection separately. The incidence of any infection was higher during the post-exposure period compared with the control periods. In this study, one patient developed osteomyelitis during the post-exposure period: a 71-year-old woman who received a betamethasone injection (14 mg/2 mL) into the soft tissue medial to the knee and was hospitalized seven days later due to osteomyelitis of the L4 vertebra. Another patient developed osteomyelitis during the late control period: a 92-year-old woman with a chronic, complicated diabetic ulcer on her left leg that later involved the bone. Two patients developed septic arthritis during the post-exposure period: a 60-year-old man who received a betamethasone injection (14 mg/2 mL) into the knee was hospitalized eight days later with septic arthritis of the same knee; and a 48-year-old man who received a depomedrol injection (7 mg/1 mL) into a finger was diagnosed with septic arthritis of another finger 77 days later. The authors concluded that intra-articular and soft-tissue corticosteroid injections may be associated with an increased risk of infection; however, the absolute risk increase appears to be low, especially if precautions are taken (e.g., sterile gloves, accurate skin disinfection, no-touch techniques). Another study by Xu et al. investigated deep knee infection, which includes septic arthritis and chronic low-grade infection as possible complications following intra-articular injections [109]. Xu et al. analyzed 50 patients with IA injection–induced deep knee infection who underwent surgical treatment between January 2010 and May 2016. These cases were matched in a 1:5 ratio with non-infected controls who had also received IA injections, matched by age, sex, and date of admission. All intra-articular injections (for both cases and controls) were performed within six months before admission, either at the same institution or a referring one. The authors concluded that strict training in aseptic technique is essential before physicians perform invasive knee procedures. Additionally, chronic low-grade infection following intra-articular injections is often underdiagnosed, posing a potential risk for periprosthetic joint infection after total knee arthroplasty (Table 9).

## 12. Corticosteroid-Induced Flares

Although not as dangerous as septic arthritis, a corticosteroid flare reaction (e.g., an initial worsening of symptoms, such as increased pain, swelling, warmth, and redness, that can occur shortly after CSI) is more common and can cause significant discomfort and symptoms [110]. The exact etiology of the flare response has not been determined, but it has been proposed that either the corticosteroid crystals themselves or the rapid intracellular uptake of microcrystalline corticosteroid esters may trigger the reaction [111]. This response has been observed with various corticosteroid formulations. Studies involving sample sizes ranging from 11 to 1754 patients have reported an incidence between 2% and 50%, with corticosteroid flare reaction being the most frequently noted side effect of corticosteroid injections. In the study by Goldfarb et al., patients with trigger finger or de Quervain’s tenosynovitis were treated with either a combination injection of methylprednisolone, lidocaine, and bupivacaine (57 patients) or a balanced pH formulation including bicarbonate (68 patients). There was no significant difference between the two groups in the rate of flare reactions (31.6% and 33.8%, respectively) [112]. Similarly, Price et al. reported no difference in flare response between two corticosteroid formulations (hydrocortisone and triamcinolone) or between two doses of triamcinolone [113]. Distinguishing between a corticosteroid flare and septic arthritis can be challenging. Corticosteroid flares tend to occur earlier than septic arthritis following an injection and resolve spontaneously. A flare typically develops within the first or second day. For instance, Berger and Yount described a rare case of early-onset corticosteroid flare in a 32-year-old man with right knee pain who received an intra-articular injection and returned 90 min later with severe pain. A second injection did not relieve the pain, which improved only after 24 h of treatment with anti-inflammatory medications and bed rest. In terms of duration, flare reactions may begin to subside within an hour, but they have been reported to last as long as 4, 7, or even 10 days [114] (Table 10).

## 13. Importance of US-Guided Injections for Pharmacological Aspect of Corticosteroids

In musculoskeletal rehabilitation, the use of image-guided techniques, particularly ultrasound-guided (US-guided) injections, has emerged as a critical best practice for delivering corticosteroids with maximal safety and efficacy. Several systematic reviews and meta-analyses demonstrate that US-guided intra-articular injections consistently achieve higher accuracy than traditional landmark-guided approaches across a wide range of joints, including the knee, hip, shoulder, wrist, and small peripheral joints [115,116,117]. For instance, in knee injections, the success rate for accurate intra-articular placement increases from approximately 77–83% with anatomical guidance to 95–96% with ultrasound guidance (*p* < 0.001) [1]. Similarly, pooled data for hip injections report near-complete accuracy with ultrasound guidance (≈100%; 95% CI 98–100%), compared with substantially lower rates for landmark-based techniques (≈72%; 95% CI 56–85%) (*p* < 0.0001) [116]. This improvement in accuracy is not merely technical but carries significant pharmacological implications, particularly when administering particulate corticosteroids such as triamcinolone acetonide or methylprednisolone acetate, whose therapeutic efficacy depends on precise intra-articular deposition and slow, local dissolution [25]. Because these formulations consist of poorly soluble microcrystalline suspensions that create a sustained intra-articular drug depot, accurate placement is essential to optimize local pharmacokinetics, maximize intra-articular drug bioavailability, and maintain prolonged anti-inflammatory activity. Conversely, inaccurate needle placement may result in extra-articular deposition or unintended distribution into peritendinous, muscular, capsular, or subcutaneous tissues, thereby compromising the intended depot effect and reducing therapeutic efficacy. This consideration is particularly relevant for particulate corticosteroids because prolonged crystal persistence within non-target tissues may increase local corticosteroid exposure and has been associated with subcutaneous fat atrophy, skin hypopigmentation, tendon degeneration, tendon rupture, and post-injection steroid flare [35,37,116].

Ultrasound guidance enables real-time visualization of the needle trajectory, joint capsule, synovial recesses, and surrounding anatomical structures (e.g., tendons, bursae, neurovascular bundles), thereby enhancing procedural safety and minimizing iatrogenic injury. This is particularly relevant in anatomically complex regions such as the hip or shoulder, where blind injections are associated with greater variability and risk [116,117].

Although direct evidence demonstrating that US guidance significantly reduces overall adverse-event rates remains limited, the consistently higher procedural accuracy achieved with ultrasound is expected to reduce inadvertent extra-articular corticosteroid deposition and unnecessary exposure of periarticular soft tissues. Consequently, US guidance may reduce the likelihood of local corticosteroid-related complications while preserving optimal intra-articular drug bioavailability [93,117].

Importantly, evidence from randomized controlled trials and systematic reviews indicates that US-guided corticosteroid injections not only improve procedural accuracy but also provide superior short-term clinical outcomes for several musculoskeletal disorders. Compared with landmark-guided injections, US guidance has been associated with greater pain reduction, improved functional recovery, higher patient satisfaction, and a reduced need for repeat procedures during the first 6–12 weeks following treatment [93,116,117].

From a cost-effectiveness and patient safety perspective, ultrasound guidance reduces the likelihood of failed injections and the need for repeat procedures—an especially relevant consideration when using high-potency or long-acting corticosteroids, whose cumulative systemic exposure and local tissue effects may increase with repeated administration [22,25]. Furthermore, inter-individual variability in joint anatomy (e.g., body habitus, presence of effusion, osteophytes) further supports the use of imaging guidance to ensure optimal drug delivery. Taken together, these findings emphasize that the clinical performance of injectable corticosteroids depends not only on their pharmacodynamic and pharmacokinetic properties but also on the precision with which they are delivered to the intended anatomical target. Accurate US-guided administration maximizes intra-articular drug deposition while minimizing inadvertent extra-articular distribution into periarticular soft tissues, thereby optimizing local drug bioavailability and the sustained-release characteristics of depot corticosteroid formulations with accurate drug delivery to maximize therapeutic efficacy while minimizing procedural and pharmacological risks.

## 14. Conclusions and Future Perspectives

Several potential side effects related to the administration of corticosteroids emerged; Table 11 reports a summary of the most prominent ones.

In light of the pharmacological and procedural considerations discussed, the integration of ultrasound guidance into corticosteroid injection therapy represents a critical step toward a more precision-oriented and rehabilitation-centered approach to musculoskeletal care. The effectiveness and safety of corticosteroids are not solely determined by their molecular structure or pharmacokinetic profile, but by the interaction between pharmaceutical formulation, physicochemical properties, image-guided delivery, and rehabilitation strategy in which they are applied. This concept is particularly relevant for particulate, long-acting formulations, whose therapeutic benefit depends on accurate localized deposition and sustained release. Within a rehabilitation framework, this precision becomes even more relevant. Corticosteroid injections are rarely a standalone intervention; rather, they are typically embedded within a broader therapeutic strategy that includes mechanical loading, functional retraining, and tissue adaptation. Inaccurate delivery may not only reduce pharmacological efficacy but also interfere with rehabilitation outcomes by altering tissue response, delaying recovery, or increasing the risk of complications. Conversely, accurate intra-articular or peri-articular targeting, facilitated by ultrasound, enhances the likelihood of achieving the desired anti-inflammatory effect while preserving the integrity of surrounding tissues critical for functional recovery. Future perspectives should therefore emphasize the integration of pharmacology, imaging, and rehabilitation sciences, moving toward standardized protocols that combine drug selection, image-guided delivery, and individualized rehabilitation programs. In this context, ultrasound-guided injection should be consistently favored for CSI, as real-time visualization of the needle, anatomical target, and surrounding structures provides greater procedural control and supports accurate drug delivery. Its relevance is particularly pronounced when using potent or particulate corticosteroid formulations and when targeting anatomically complex or high-risk regions, while the magnitude of the expected clinical and safety benefits should be interpreted according to the anatomical site, clinical setting, procedural complexity, and strength of the available evidence.

Accordingly, future research should move beyond comparisons among corticosteroid molecules alone and instead investigate formulation-specific treatment algorithms integrating physicochemical properties, anatomical target, ultrasound-guided delivery and individualized rehabilitation protocols. Such a formulation-oriented strategy may represent the next step toward precision musculoskeletal rehabilitation. Ultimately, aligning molecular pharmacology with precise delivery and structured rehabilitation may represent the most effective strategy to maximize therapeutic benefit while minimizing risk in modern musculoskeletal practice.

## Figures and Tables

**Table 1:** Comparison of the physicochemical, pharmacokinetic and pharmacodynamic characteristics of commonly used injectable corticosteroid formulations used in rehabilitation. Relative glucocorticoid potency is expressed with hydrocortisone as the reference (1×).

Parameter	Hydrocortisone Acetate	Methylprednisolone Acetate	Triamcinolone Acetonide	Betamethasone Sodium Phosphate	Betamethasone Acetate	Betamethasone Dipropionate	Dexamethasone Sodium Phosphate
**Solubility**	Moderate	Low (particulate)	Very low (particulate)	High (soluble)	Low	Very low	High (soluble)
**Structural modification**	C21 acetate ester	C21 acetate ester	C16–C17 acetonide bridge	C21 phosphate ester	C21 acetate ester	C17 and C21 dipropionate esters	C21 phosphate ester
**Lipophilicity**	Moderate	Moderate-high	High	Low	Moderate-high	High	Low
**Crystal formation**	Minimal	Microcrystals	Dense microcrystals	None	Polymorphic microcrystals	Dense microcrystalline suspension	None
**Onset of action**	Intermediate	Delayed	Delayed (24–72 h)	Rapid (hours)	Delayed	Delayed	Rapid
**Duration**	Short	Intermediate-long	Long (weeks)	Short (days–weeks)	Intermediate (~7–8 days)	Long (~16–18 days)	Short
**Synovial persistence**	Low	Moderate-high	High	Low	Moderate-high	High	Low
**Systemic peak**	Moderate	Moderate	Low–moderate	High (early peak)	Low–moderate	Low	High
**HPA suppression**	Minimal	Prolonged	Prolonged, mild	Transient	Prolonged	Prolonged	Transient
**Relative glucocorticoid p otency (Hydrocortisone ≈ 1×)**	1×	5×	5×	25–30×	25–30×	25–30×	25–30×
**Risk of flare**	Low	Moderate	Higher	Low	Low	Low	Low
**Preferred indication**	Mild inflammatory conditions	Large joints, chronic inflammatory disorders	Large joints, chronic OA	Acute inflammatory flares	Acute inflammation, peritendinous and perineural injections	Acute inflammation, tendinopathic/peritendinous pain, dermatologic inflammatory conditions	Acute inflammation, peritendinous and perineural injections
**Ultrasound appearance**	Slightly echogenic	Echogenic	Echogenic particulate cloud	Anechoic	Anechoic to slightly echogenic (particulate)	Anechoic to slightly echogenic (particulate)	Anechoic

**Table 2:** Potential chondrotoxic effects of corticosteroids.

Key Outcomes	Involved Corticosteroid(s)	Evidence (Reference)	Human/In Vitro/Animal
**Accelerated progression of Osteoarthritis**	All corticosteroid classes	The acceleration is characterized by a rate of joint space loss that exceeds the typical progression observed over a short timeframe (Wernecke et al. (2015) [26]; Hochberg (2015) [27]; Kamel et al. (2024) [28])	Human
**Elevated probability of radiographic OA progression in the knee**	All corticosteroid classes	The probability seems 4.67 times higher in patients who receive continuous CSI (Zeng et al. (2019) [29])	Human
**Detrimental degradative effects on articular cartilage, specifically interference with protein synthesis (proteoglycans, type II collagen, and aggrecan)**	Methylprednisolone acetate, Dexamethasone sodium phosphate, Betamethasone sodium phosphate	Associated with high doses (>3 mg/dose or 18–24 mg/dose cumulative total) and prolonged exposure. (Wernecke et al. (2015) [26])	Human
**Reduced cell viability, cartilage damage, inhibition of extracellular matrix (ECM) synthesis, reduced proteoglycan levels and enhanced toxicity when combined with lidocaine**	Methylprednisolone acetate, Dexamethasone sodium phosphate, Betamethasone sodium phosphate	Demonstrated toxicity in vivo (in rabbits and dogs) and in vitro, though some found no adverse effects or suggested potential structure-modifying activity (Pirri et al. (2024) [31])	Animal and in vitro
**Radiographic progression of hip OA**	Triamcinolone acetonide (40 mg/4 mL 0.5%)	Progression observed in 44% of patients in a study following patients post-procedure for an average of 6 months (Simeone et al. (2019) [30])	Human
**Significantly greater reduction in cartilage volume compared to placebo, suggesting risk of accelerated cartilage degeneration.**	Triamcinolone acetonide (40 mg every three months for two years)	Demonstrated in clinical trials of repeated intra-articular injections (McAlindon et al. (2017) [24]; Bharadwaj et al. (2025) [36])	Human
**Mixed findings, including no damage or protective effects on chondrocytes.**	Triamcinolone acetonide	Experimental and clinical evidence shows mixed results regarding chondrotoxicity (Pirri et al. (2024) [31])	Human
**Significant and consistent chondrotoxicity**	Betamethasone acetate	Both after exposure for the mean duration of action (approximately 9 days) and in a 14-day time-controlled trial, with cell mortality exceeding 20% (Dragoo et al. 2012 [32])	In vitro
**Amplified chondrotoxic effect**	Betamethasone acetate plus local anesthetics	Combination of betamethasone with 1% lidocaine or 0.25% bupivacaine causes chondrocyte mortality of 76% and 66% at 14 days (Braun et al. 2012 [33])	In vitro

**Table 3:** Relation between calcifications and corticosteroids.

Key Outcomes	Involved Corticosteroid(s)	Evidence (Reference)	Human/In Vitro/Animal
**Development of dystrophic calcification**	Corticosteroids (Low water solubility preparations, often extra-articular)	Calcifications are typically pericapsular or intracapsular. Caused by local tissue damage from the needle and the low water solubility of the extra-articular corticosteroid, leading to chronic granulomatous inflammation (Gilsanz and Bernstein (1984) [37]; Habib et al. (2010) [38])	Human
**Risk of intra-articular calcification**	Triamcinolone hexacetonide	Reported in the context of intra-articular CSI for chronic OA in children; youth and joint size are possible predisposing factors (Job-Deslandre and Menkes (1990) [40]; Isoda et al. (2021) [41])	Human
**Synovial cyst calcification**	Prednisone	Documented in a 57-year-old man following an intra-facet joint injection, with subsequent calcification observed six months post-injection (Boissière et al. (2013) [43])	Human

**Table 4:** Relation between tendinopathy and corticosteroids.

Key Outcomes	Involved Corticosteroid(s)	Evidence (Reference)	Human/In Vitro/Animal
**Suppression of human tenocyte cellular activity and collagen production, leading to altered tendon structure and potential spontaneous rupture**	Triamcinolone acetonide	Triamcinolone has been hypothesized to directly harm human tenocytes by suppressing cellular activity and collagen production (Tempfer et al. (2009) [46])	Human
**Significant reduction in biomechanical strength of Achilles tendons (in vivo rat study), characterized by collagen attenuation, increased expression of MMP-3, and apoptotic cells**	Triamcinolone acetonide, Prednisolone	Both corticosteroids significantly reduced the biomechanical strength in corticosteroid-treated groups (Muto et al. (2014) [51])	Animal
**Decreased proliferation rates and collagen synthesis in rotator cuff tendons; promotion of adipocyte and chondrocyte differentiation**	Triamcinolone acetonide	MMP2, MMP9, and TIMP1 expression modulation (Muto et al. (2013) [52])	In vitro
**Inhibition of tendon cell migration, cell proliferation, and reduced collagen synthesis**	Dexamethasone (form not specified)	Inhibition of cell migration is correlated with reduced alpha-smooth muscle actin gene expression. An initial inhibitory effect on Achilles tendon fibroblasts was observed at a concentration of 10^−4^ M (Tsai et al. (2002) [50])	Animal
**Risk of localized necrosis and degenerative changes if injected directly into tendon tissue, potentially weakening tensile strength**	Corticosteroid microcrystals (e.g., Triamcinolone acetonide, Methylprednisolone)	Caused by the precipitation of microcrystalline suspensions designed for sustained release (Speed (2001) [53]; Fredberg (1997) [54]; Fackler et al. (2023) [55])	In vitro
**Lower likelihood of persistence and accumulation compared to other preparations**	Betamethasone sodium phosphate/betamethasone acetate	Its crystal size is usually smaller, and it is less particulate than triamcinolone acetonide or methylprednisolone (Benzon et al. (2007) [56]; Cole and Schumacher (2005) [57])	In vitro
**Collagen-synthesis suppression and tendon rupture**	Betamethasone sodium phosphate plus poorly soluble dipropionate/acetate ester	Injections performed within six months of tendon repair surgery or with repeated dosing (Kamel et al. (2024) [28])	In vitro

**Table 5:** Potential corticosteroid-related osteonecrosis.

Key Outcomes	Involved Corticosteroid(s)	Evidence (Reference)	Human/In Vitro/Animal
**Impairment of blood flow/bone perfusion due to vascular obstruction**	All Corticosteroid Classes	Corticosteroids alter lipid metabolism, causing swelling and necrosis of adipocytes, increased lipid deposition in osteocytes, hyperlipidemia, fat embolism, and fatty infiltration of the bone marrow (Kim and Ko (2023) [66]; Birla et al. (2021) [67])	Human
**Increased bone cell death**	All Corticosteroid Classes	Corticosteroids can induce apoptosis of bone cells via pathways such as the Fas pathway (Wang et al. (2013) [65])	In vitro
**Damage to vessels and formation of blood clots**	All Corticosteroid Classes	Steroids can damage vascular endothelial cells and promote a hypercoagulable state, increasing the risk of thrombus formation and further reducing blood supply to bone (Kerachian et al. (2009) [68]; Xie et al. (2015) [69])	Human

**Table 6:** Potential cutaneous side effects of corticosteroids.

Key Outcomes	Involved Corticosteroid(s)	Evidence (Reference/Context)	Human/In Vitro/Animal
**Cutaneous atrophy (skin thinning) and subcutaneous fat atrophy**	All corticosteroid classes	Caused by loss of subcutaneous adipose tissue and decreased Type I and Type III collagen synthesis, resulting in dermal thinning (Jang et al. (2011) [79])	Human
**Hypopigmentation or depigmentation (skin whitening)**	Triamcinolone acetonide	Clinically presents alongside atrophy as atrophic macules, often displaying a linear or branching distribution. Hypopigmentation is mainly attributed to melanocyte dysfunction (Porcar Saura et al. (2022) [76]; Park et al. (2013) [84])	Human
**Persistent soft tissue changes**	Less soluble, longer-acting corticosteroid preparations (e.g., Triamcinolone acetonide, Methylprednisolone)	The low solubility of these compounds causes persistence in the tissues (Shah et al. (2019) [87])	Human
**Reversible fat atrophy**	All corticosteroid classes	Subcutaneous fat atrophy is generally reversible, resolving within 6–12 months post-injection (Kim et al. (2015) [85])	Human
**Hypopigmentation**	Betamethasone dipropionate	Wrist injections in female patients, onset between 4 and 10 weeks, complete resolution in 7.14 months on average (Weinstein et al. (2025) [91])	Human
**Subcutaneous fat atrophy**	Betamethasone dipropionate	Rarer (6.7% of cases), incomplete recovery in two out of three cases even after 18 months of follow-up (Weinstein et al. (2025) [91])	Human

**Table 7:** Corticosteroid-related muscle atrophy.

Key Outcomes	Involved Corticosteroid(s)	Evidence (Reference/Context)	Human/In Vitro/Animal
**Severe localized muscle atrophy following a single injection (rare occasion)**	Triamcinolone acetonide (20 mg)	A case of a young woman who developed severe muscle atrophy in the wrist after a single injection of 20 mg into the transverse carpal ligament was reported (Park et al. (2013) [84])	Human
**Increase in the expression of muscle atrophy-related genes.**	Corticosteroid Injections (CSIs) (General)	Pre-clinical data suggest that frequent CSI could increase the expression of detrimental genes, such as muscle atrophy-related genes (Lee et al. (2024) [100])	Human

**Table 8:** Corticosteroid-related nerve lesions.

Key Outcomes	Involved Corticosteroid(s)	Evidence (Reference/Context)	Human/In Vitro/Animal
**Pain and numbness in the lateral aspect of the foot, resulting in damage to the lateral plantar nerve**	Methylprednisolone acetate	Reported in a case of a 41-year-old man following an injection for plantar fasciitis (Snow et al. (2005) [107])	Human
**Severe median nerve injury**	Triamcinolone acetonide	Documented in a case report following a local injection for Carpal Tunnel Syndrome (Zhou and Lu (2020) [105])	Human

**Table 9:** Risk of infection related to corticosteroid administration.

Key Outcomes	Involved Corticosteroid(s)	Evidence (Reference/Context)	Human/In Vitro/Animal
**Increased incidence of any infection during the post-exposure period compared with control periods**	Intra-articular and Soft-Tissue CSI	Population-based study (15,732 adults) found that CSI may be associated with an increased risk of infection	Human
**Development of septic arthritis of a finger 77 days post-injection (case report).**	Methylprednisolone acetate (7 mg/1 mL)	Case of a 48-year-old man who received an injection into a finger (Zacay and Heymann (2023) [108])	Human
**Development of osteomyelitis of the L4 vertebra seven days post-injection**	Betamethasone (14 mg/2 mL, form not specified)	Case of a 71-year-old woman who received an injection into the soft tissue medial to the knee (Zacay and Heymann (2023) [108])	Human
**Development of septic arthritis of the knee eight days post-injection**	Betamethasone (14 mg/2 mL, form not specified)	Case of a 60-year-old man who received an injection into the knee (Zacay and Heymann (2023) [108])	Human
**Risk of local infection after injection due to physical shielding.**	Corticosteroid microcrystals	Microcrystals can physically shield bacteria from immune defenses in the area (Fackler et al. (2023) [55])	In vitro

**Table 10:** Corticosteroid-induced flares.

Key Outcomes	Involved Corticosteroid(s)	Evidence (Reference/Context)	Human/In Vitro/Animal
**High incidence rate of the flare reaction.**	Corticosteroid crystals or microcrystalline corticosteroid esters	The reaction may be triggered by the corticosteroid crystals themselves or the rapid intracellular uptake of microcrystalline corticosteroid esters (Honcharuk and Monica (2016) [111])	Human
**Flare rate comparison between different formulations**	Methylprednisolone acetate (combined with lidocaine and bupivacaine) vs. balanced pH formulation	Goldfarb et al. found no significant difference in the rate of flare reactions between two groups (31.6% and 33.8%, respectively) (Goldfarb et al. (2007) [112])	Human
**Flare rate comparison between different formulations and doses**	Hydrocortisone and Triamcinolone acetonide (and two doses of Triamcinolone)	Price et al. reported no difference in flare response between the two corticosteroid formulations or between two doses of triamcinolone (Price et al. (1991) [113])	Human

**Table 11:** Comparison of injectable corticosteroid formulations: adverse effects, typical local injectable doses and current areas of uncertainty. * Typical adult doses reported for musculoskeletal local injections; actual dose depends on the anatomical site, target tissue and clinical indication.

Specific Glucocorticoid Formulation	Associated Side Effects	Typical Injectable Dose *	Grey Areas/Uncertainties
**Hydrocortisone acetate**	Skin atrophy; transient local pain; lower incidence of tissue atrophy; systemic adverse effects uncommon	5–25 mg	Limited contemporary evidence because this formulation is less frequently used in musculoskeletal injections.
**Methylprednisolone acetate**	Skin atrophy; hypopigmentation; tendon rupture; dose-dependent chondrotoxicity; calcification; osteonecrosis; transient systemic corticosteroid effects	20–80 mg	Conflicting evidence regarding cartilage toxicity. Limited data defining safe cumulative dose and long-term outcomes after repeated injections.
**Triamcinolone acetonide**	Skin atrophy; hypopigmentation/depigmentation; tendon weakening or rupture; subcutaneous fat atrophy; post-injection flare; potential chondrotoxicity; calcification; rare muscle atrophy; nerve injury; osteonecrosis (high cumulative exposure)	10–40 mg	Mechanisms of hypopigmentation remain unclear. Conflicting evidence regarding chondrotoxicity and no consensus on safe cumulative dose or injection frequency.
**Betamethasone sodium phosphate**	Skin atrophy; hypopigmentation; tendon injury; possible chondrotoxicity; muscle atrophy after prolonged exposure; osteonecrosis (rare)	2–6 mg	Clinical evidence varies according to formulation. Some studies use combined phosphate/acetate preparations, limiting direct comparison with pure sodium phosphate.
**Betamethasone acetate**	Chondrotoxicity; dystrophic calcification; lower tendon-rupture risk; collagen-synthesis suppression; skin hypopigmentation and subcutaneous fat atrophy	3–6 mg	Most clinical data derive from compound formulations (acetate + sodium phosphate), limiting isolation of acetate-specific effects; in vitro chondrotoxicity findings not yet confirmed in long-term human cohorts
**Betamethasone dipropionate**	Dystrophic calcification; tendon collagen-synthesis suppression and rupture risk; skin hypopigmentation; muscle atrophy not detected in short-term RCTs; no adverse bone events reported	5–10 mg	Long genomic activity vs. short plasma half-life raises uncertainty about true duration of tissue exposure; hypopigmentation/atrophy risk appears strongly site-dependent (wrist vs. elbow vs. knee) with no consensus on anatomical risk stratification; osteonecrosis and muscle atrophy data limited to small, short-duration trials
**Dexamethasone sodium phosphate**	Skin atrophy; hypopigmentation; tendon weakening; experimental chondrotoxicity; extracellular matrix suppression; muscle atrophy after prolonged exposure	2–8 mg	Most evidence derives from experimental studies; translation of laboratory findings to clinical practice remains uncertain.

## Data Availability

No new data were created or analyzed in this study. Data sharing is not applicable to this article.
